# Analyzing the role of family support, coping strategies and social support in improving the mental health of students: Evidence from post COVID-19

**DOI:** 10.3389/fpsyg.2022.1064898

**Published:** 2022-12-23

**Authors:** Chunying Yang, Hong Gao, Yuxiang Li, Enguo Wang, Nina Wang, Qinglei Wang

**Affiliations:** ^1^School of Special Education, Zhengzhou Normal University, Zhengzhou, Henan, China; ^2^Faculty of Education, Henan University, Kaifeng, Henan, China; ^3^Department of Educational Psychology and Counselling, Faculty of Education, University of Malaya, Kuala Lumpur, Malaysia; ^4^Faculty of Sports and Exercise Science, University of Malaya, Kuala Lumpur, Malaysia

**Keywords:** coping strategies, family support, social support, mental health, COVID-19

## Abstract

**Background:**

The COVID-19 pandemic and the multifaceted response strategies to curb its spread both have devastating effects on mental and emotional health. Social distancing, and self-isolation have impacted the lives of students. These impacts need to be identified, studied, and handled to ensure the well-being of the individuals, particularly the students.

**Aim:**

This study aims to analyze the role of coping strategies, family support, and social support in improving the mental health of the students by collecting evidence from post COVID-19.

**Methods:**

Data was collected from deaf students studying in Chinese universities of Henan Province, China. A survey questionnaire was designed to collect data from 210 students. Descriptive statistics were calculated using SPSS 21 while hypothesis testing was carried out using Mplus 7.

**Results:**

The results demonstrated that family support was strongly positively linked to mental health and predicted coping strategies. The direct relationship analysis showed that coping strategy strongly predicted mental health. Furthermore, coping strategies significantly mediated the relationship between family support and mental health. Additionally, the results highlighted that PSS significantly moderated the path of family support and coping strategies only.

**Conclusion:**

Family support and coping strategies positively predicted mental health, whereas, family support was also found to be positively associated with coping strategies. Coping strategies mediated the positive association between family support and mental health. However, perceived family and other support only moderated the relationship between family support and coping strategies.

## 1. Introduction

Mental health has become a topic of increased concern in recent years in COVID-19. Numerous studies have provided evidence suggesting heightened levels of psychological distress in this community compared to the general population. This psychological strain of COVID-19 among teenagers has brought severe and long-lasting consequences of mental health, which lead to poor physical health outcomes, such as the rise of cardiovascular disorders, and poor mental health (MH) outcomes. Teenagers struggle more than adults do with the mental burden of this influence since they lack the adult’s coping mechanisms and physiological development (Rawat and Sehrawat, 2021). Teenagers who already have MH problems face even more MH issues during times of crisis (Gavin et al., 2020).

The Director General of the World Health Organization, on the recommendation of the Emergency Committee, declared the novel Corona virus also known as COVID-19 a Public Health Emergency of International Concern, and the virus has had devastating effects on the economy, education, and society (Priya et al., 2021). From the very beginning of the outbreak, health officials devised a multifaceted response strategy to stop the spread of COVID-19. A key component of the plan was self-isolation or home quarantine. Social isolation is one of the steps the governments have taken to curb the virus’s spread. Social isolation can have an impact on MH, escalating signs of stress, anxiety, and depression (Robb et al., 2020). In recent years, colleges and universities have seen an increase in the number of students experiencing psychological distress. The underlying cause might be the inexperience of college students, who frequently struggle to cope with stress, particularly when faced with academic, interpersonal, and career-related problems. Numerous studies have indicated that students from both eastern and western China have displayed symptoms of depression in the wake of COVID-19 (Chen et al., 2021; Zhang et al., 2021; Zhao et al., 2021). Numerous studies continue to place a strong emphasis on the MH of college students, particularly when it comes to identifying the underlying causes of psychopathological symptoms. Studies have also looked at how students’ transition to university is impacted by academic stress. These research found that undergraduate students were already more stressed, anxious, and depressed than the overall population (Guo et al., 2021). The already poor prospects for these students’ MH were further aggravated by the stress and worry associated with COVID-19.

These factors are connected to distinct socially isolating situations, such as classroom distractions, the use of particular teaching techniques that are socially isolating, and the overwhelming uncertainty that resulted from precautionary measures to stop or slow the spread of the virus have also added to the severity of the situation. The general public along with particular circumstances that may be having various effects as a result of the actions performed during this time, amplifying its magnitude, has been studied in various studies undertaken in the pandemic context (Covan, 2020; Scharmer et al., 2020; Bakioğlu et al., 2021; Wu et al., 2021). The majority of studies conducted with undergrads during the disease outbreak have concentrated on MH; understanding of knowledge and traits associated with the pandemic; and how educational strategies were crafted to cope with the challenge (Gavin et al., 2020; Liang et al., 2020; Pfefferbaum and North, 2020; Budimir et al., 2021; Saltzman et al., 2021). Other research has noted the rise in stress, sadness, and anxiety symptoms as well as the coping mechanisms of undergrads (Kobbin et al., 2020). Undergraduate students may experience new or increased stress due to the COVID-19 pandemic, which can be upsetting and result in a variety of MH issues.

The general lack of clarity surrounding the pandemic, the abrupt switch to and participation in online classes, and COVID-19’s effects on the lives of students were frequent causes of heightened stress and worry (Rodríguez-Hidalgo et al., 2020). According to a study, the COVID-19 outbreak puts students at a greater risk of acquiring depression and suicidality (Sinyor et al., 2021). After university campuses closed, students continued to perceive their academic future as having relatively dim prospects. The situation was rendered even more precarious for students by social isolation and a lack of sufficient and efficient MH support. Due to these variables, young people who are attending college are now even more at risk of developing MH problems (Li X. et al., 2021).

Social support is defined as the provision of practical assistance by members of extended family, friends, and coworkers (Bjørlykhaug et al., 2021). Likewise, it is defined as “support made available to an individual through social relationships to other people, groups, and the greater community” (Heaney and Israel, 2008). There are multiple ways to measure social support, however, one of the most commonly used indexes of social support has been perceived social support (PSS). It is not only easy to measure the PSS but the accuracy aspect is also of utmost importance as it depicts a relatively precise state of MH. Numerous studies have shown how PSS improves MH in stressful circumstances (Wang and Eccles, 2012). Signs of deteriorating MH have been observed in people who receive less or no social support when they are stressed and depressed (Li F. et al., 2021). PSS also has an impact on the course of depression and is crucial to recovery from affective disorders. Coping is the term for people’s ways of thinking and acting to deal with the internal and external pressures of stressful situations (Kobbin et al., 2020).

Studies have demonstrated how various groups adopt coping mechanisms, a central idea in psychology research, with particular attention to the issue at hand (Budimir et al., 2021). People with a high intolerance for uncertainty were more likely to perceive the pandemic as dangerous and to employ coping techniques that centered on emotions during prior epidemic occurrences, such as the H1N1 pandemic in 2009. This was revealed by research on stress management in epidemic events (Vogel et al., 2022). Research with university students during the SARS pandemic in 2003 highlights the importance of coping as a defense mechanism against the damaging effects of stressors on overall perceived health (Blume et al., 2021). Additionally, research revealed that in response to stressors connected to SARS, people used less active coping strategies (focused on problems) and more avoidable coping mechanisms (focused on emotions; Bai et al., 2004). Adaptive strategies in the wake of the pandemic can be explored, particularly with regard to the educational setting, such as the substitution of distant activities for on-site activities. A key factor influencing depression symptomatology is social support. Numerous studies have demonstrated that people who are depressed experience considerable, persistent psychosocial challenges.

Particularly, lower levels of social support are linked to higher levels of depression symptoms (Wang and Eccles, 2012). The standard of emotional support offered by others is referred to as PSS. Additionally, studies reveal that levels of PSS are strongly linked to metrics of lowered stress and mental distress along with enhanced well-being (Wang et al., 2018). However, the majority of studies on adolescents’ social support focus on their families, and very few studies look at their peers’ social support (Oktavia et al., 2019). Our study takes into account how family, friends, and significant others are seen in terms of social support. According to a study of the literature and research, social support (particularly as perceived by adolescents) has been examined both separately as a psychological and social risk factor for depression and in conjunction with other factors to moderate the relationship between life events and depression, or as a moderator variable, as it is in this study. One of them is the buffering model, which holds that support shields people from the potentially harmful consequences of stressful situations (MulejBratec et al., 2020). This study sought to ascertain whether there is a connection between students’ coping mechanisms during social withdrawal brought on by the pandemic and MH based on the gap in current knowledge. The research also looked at how family support (FS) and PSS (PSS) are associated with the mental health (MH) of the students. Furthermore, it investigates the mediating role of coping strategies and moderating role of PSS. It is anticipated that the findings of this inquiry will advance understanding in these areas.

Even though there have been numerous studies in the field of MH, however, the literature gap regarding the roles and relationships of coping strategies and PSS, as well as their mediating and moderating behaviors still exists. The goal of this research is to create a framework for concurrently examining the roles and interactions of the variables. The following research questions are the focus of the study.

How does FS influence the MH of the students after COVID-19? How FS is associated with coping strategy? How do coping strategies influence the MH of students post-COVID-19? What role is played by coping strategies between FS and MH? How does PSS from friends and others affect the relationship between FS and MH? How does PSS from friends affect the relationship between FS and coping strategy?

After an extensive review of available relevant literature, it was found that studies have been done to investigate coping strategies and MH related but no study has been done until now to know the answers to these research questions using a single framework. Thus, from the available literature, the current study firstly assumes FS positively influences MH and is positively associated with a coping strategy. The study further assumes that coping strategy positively influences MH and it positively mediates the relationship between FS and MH. It is also hypothesized in the current study that PSS from friends and others positively moderates the relationship between FS and MH and PSS from friends and others positively moderates the relationship between FS and coping strategy.

The following are the potential contributions of the study: Firstly, in accordance with the stress-health theory, the study offers a thorough and systematic analysis of the ideas of coping mechanisms, FS, and MH. Secondly, the study makes a contribution through its creative and distinctive approach, which suggests and examines the connections between PSS and coping mechanisms. Thirdly, the research adds to the integrated analytical framework that examines the connection between familial support, coping mechanisms, and MH by incorporating PSS from friends and other people. The theoretical perspective is explicated in an original form by the study’s framework. Additionally, the work has both applied as well as theoretical implications. Following is a summary of the remaining section of the current study: The overview of the literature was offered in Section 2 of this research study. In Section 3, actual techniques and analyses have been presented. The statistical analysis and empirical findings were reported in the following section. The debate, findings, and theoretical and practical ramifications have all been condensed at the end.

## 2. Literature review and hypotheses development

### 2.1. Theoretical support and background

According to the conservation of resource theory, stressful situations could lead to difficulties with a person’s physical or MH (Mao et al., 2021). The COVID-19 pandemic’s primary characteristics as a novel infectious illness are that it is extremely contagious and dangerous, developing quickly, and has no effective medications for either prevention or therapy. Students’ MH during the pandemic outbreak was impacted by a number of stressful pandemic-related factors, such as personal daily routine disruptions and individual physical health under threat from the COVID-19 sickness.

Mental illness is a result of social exclusion and ongoing worry about contagion. The COVID-19 situation has had significant negative effects on people’s health (Pfefferbaum and North, 2020). However, researchers are concentrating more on how this quickly developing worldwide catastrophe may affect the MH of the aging population. The psychological impact of COVID-19 on teenage MH has gotten very little consideration (Rawat and Sehrawat, 2021). The psychological strain of COVID-19 among teenagers should be a prominent aspect in the COVID-19 study due to the severe and long-lasting consequences of MH, which lead to poor physical health outcomes, such as the rise of cardiovascular disorders, and poor MH outcomes. Teenagers struggle more than adults do with the mental burden of this influence since they lack the adult’s coping mechanisms and physiological development (Rawat and Sehrawat, 2021). Teenagers who already have MH problems face even more MH issues during times of crisis (Gavin et al., 2020).

### 2.2. Relationship between coping strategy, family support and mental health

The effects of COVID-19 on family well-being include a quarantine that restricts movement, financial difficulty, decreased income, a lack of job, obesity, and worsening non-communicable diseases. Nature and range of risks that have emerged after COVID-19 have expanded and increased. The risks involve physical as well as psychological aspects such as the risk of hospitalization or even losing one’s life, failing to access food, vulnerability related to family, and psychological health problems. Furthermore, the chances of maltreatment of children also increased in the wake of social isolation (Rapoport et al., 2021). A survey revealed increased instances of child treatment during school breaks, summer, and amid natural disasters (Rapoport et al., 2021). The only seemingly beneficial aspect of COVID-19 might be the benefit of having more family time that one could spend as the restrictions on the movement either slowed down or stopped the mobility of people confining them to their residences.

Additionally, family interactions have also been severely affected by COVID-19(Pan, 2020). In difficult times like COVID-19, a family’s ability to manage difficulties and deal with risks, stress, and crises is referred to as family support. The capacity to not only survive but also recover from the negative impact of any troublesome situation can be referred to as FS. Some key factors in developing this capacity of FS are adaptation, acceptance, and management in the wake of untoward incidents (Shin et al., 2021). Parents are essential in building the family’s resilience during a widespread public health crisis. Children’s and teenagers’ resilience is influenced by parents’ resilience, including how well they look after their extended family members while still being able to take care of themselves.

Children can adapt well to the epidemic if their parents do so in a positive way. It’s critical to have parental assistance available to lessen the effects of COVID-19. Support from family members during the epidemic may lessen concerns about depression and anxiety (Yang et al., 2020). According to studies, a large number of people experience tension and worry while there are extended lockdowns because of the pandemic (Budimir et al., 2021). The level of concern in people regarding contracting COVID-19 themselves is lower than this apprehension for their family members (Gavin et al., 2020). In addition, elderly people tend to experience less anxiousness.

Anxiety lessens with an increase in age. Core reasons lie in the fact that coping mechanisms become more and more effective with an advance in age, moreover, better financial stability also plays its part (Drentea, 2000). Adjustment, social integration, greater communication, and proper financial management are significant FS factors. Maintaining family resilience requires flexibility, which is the ability to pay attention to changing circumstances and adjust one’s approach to meet those demands.

Due to school closures caused by the COVID-19 pandemic, students now take their classes online. Physical activity, travel, and other logistical stresses may be lessened as a result of home learning. On the other hand, stress can also result from home education. Some students may find home learning to be very difficult owing to scholastic issues, limited internet connection, and insufficient resources (Soetisna et al., 2021). During the pandemic, families might lack the tools and information necessary to manage at-home education. In addition, parents struggle to raise their children while juggling domestic chores, working remotely in the wake of economic insecurity, and fear of loss of work. Parents struggle to achieve a work-life balance and contend with several shifting circumstances while engaging in physical distance, which increases stress (Dinh, 2020).

Families experience distress when schools are closed. To boost children’s and parents’ readiness to accept homeschooling during the epidemic and ensure that kids get good grades, families have to be better educated on how to assist one another. Households with special needs children have to cope with additional stress during a pandemic. Children with special needs spend all of their time living with family members as a result of the closure of the school. Children have little time for social activities outside of their homes during the pandemic. The need for an easy environment at home becomes even more important and pertinent particularly for the family which has special needs child. In order to create a setting that fosters family resilience, parents must learn to control their stress and obtain social support. The pandemic has drastically changed the family routines that affect both physical as well as MH of family members (Ameis et al., 2020). The family can deal with the pandemic together, though, by creating routines and engaging in activities that involve all members, such as scheduling mealtimes together, participating in household chores together, fostering effective communication, engaging in thrilling activities, getting regular physical workouts, being vigilant regarding cleanliness, and developing good sleep habits, which is crucial, particularly in the wake of COVID-19.

These habits may enhance the family’s capacity to care for each other and provide better support to one another. Families are better able to help one another when there are protective factors (which foster the capacity for adaptation or recovery in times of crisis) and recovery variables (which assist in development growth; Semenov and Zelazo, 2019). These factors may make the family more competent to deal with the crisis and struggle as a unit. The main factors in FS are a wholesome amalgamation of useful and healthy family activities that help a family create and maintain a strong trust-based bond enabling all family members to sustain during adverse circumstances (Semenov and Zelazo, 2019). From all the above research findings about FS, MH, and coping strategy it is assumed that FS has a positive relationship with coping strategy and MH.

*H1*: Family support is to positively influence the mental health.

*H2*: Family support is positively associated with coping strategy.

How people handle stress has a significant psychological impact on how stressful life events like COVID-19 affect people’s MH. The broad definition of coping is the mental and behavioral strategies people use to control their stress (Obbarius et al., 2021). When faced with challenging circumstances, like the pandemic, it is typical to use coping mechanisms or behaviors that encourage adjustments and solutions to the problems being faced. People employ a variety of coping mechanisms to deal with difficult situations or times (Budimir et al., 2021). The two types of coping models, according to Folkman and Lazarus, are the problem-and emotion-focused models (Obbarius et al., 2021). When faced with a stressful scenario, both coping mechanisms are employed, however, their efficacy varies. Self-distraction, stress management, exclusion, medication, moral support usage, keeping informed, and behavioral adjustments are just a few of the many coping mechanisms that have been proven extremely effective. However, the question of whether one strategy is better than another is hotly contested. More successful than other methods for reducing the effects of stress and promoting MH can be those that include addressing and resolving stresses (Nshimyiryo et al., 2021).

Avoidant tactics may be effective in lowering short-term stress, but they are typically viewed as damaging in the long run since they do not directly address the stressor, exposing people to high levels of stress over extended periods. A person’s stress response may have an impact on their health over time. It is therefore likely that coping with COVID-19-related trauma inappropriately may lead to MH issues in the future. To improve family wellbeing, it’s critical to establish a regular appreciation practice. To foster a sense of community, loyalty, harmony, and happiness among family members, it is critical that the members maintain open lines of interaction and find enjoyable activities to do regularly.

Additionally, when parents or other family members ask for assistance, forming positive reinforcement with them is crucial. Examples of these people include siblings, acquaintances, extended families, and colleagues. Technology use for support networks and interaction amid social isolation is crucial (SayinKasar and Karaman, 2021). Utilizing technological tools to avail social support helps keep friends and family connected and supportive of one another through a trying time. Resilience during the pandemic is positively impacted by supportive families, good parenting, and effective coping mechanisms. During a severe disruption scenario like a pandemic, family activities are crucial for greater well-being (Petersen et al., 2021). The above-mentioned facts from the available literature help us devise our hypotheses 3 and 4 as coping strategy influences MH positively and mediates the relationship between FS and MH.

*H3*: Coping strategy is to positively influence the mental health.

*H4*: Coping strategy to mediate the relationship between family support and mental health.

### 2.3. Social support and mental health

Social support is defined as “support made available to an individual through social relationships to other people, groups, and the greater community” (Heaney and Israel, 2008). There are multiple ways to measure social support, however, one of the most commonly used indexes of social support has been PSS. It is not only easy to measure the PSS but the accuracy aspect is also of utmost importance as it depicts a relatively precise state of MH. Numerous studies have shown how PSS improves MH in stressful circumstances (Wang and Eccles, 2012).

Though PSS and received social support are entirely distinctive from each other, however, it is generally believed that the relationship between these otherwise distinct supports should be high, particularly in scenarios when the demanded support is equivalent to the provided support (FasihiHarandi et al., 2017). Similar to this, other authors contend that the recollection of helpful behaviors displayed can be used to gauge perceived support (Özmete and Pak, 2020). We believe that social support received by an individual during the COVID-19 pandemic equals the required social support by an individual, therefore, we measured that the received social support reflected the level of PSS. However, social support and social support resources may not be mistaken as similar but they should be understood as distinct.

According to a study one’s subjective perceptions of the availability of social support from other users on social networks are represented by perceived support (Noret et al., 2020). According to various studies, perceived support quality is more closely correlated with MH than real personal network structure (Kandeğer et al., 2021). Numerous factors have an impact on people’s MH. Previous research has established that the availability of social support along with effective coping strategies helps maintain good MH (FasihiHarandi et al., 2017). A correlation between social support provided by the family and stress symptoms related to post-trauma was found. However, no such correlations were found when social support was provided by the friends. In a different study, peer social support was found to be more effective at preventing psychological distress than social support provided by family members (Özmete and Pak, 2020).

Recently, there has been a rise in interest in new approaches such as latent profile analysis (LPA) for studying the many types of networks of social support (Mai et al., 2021). Furthermore, some scholars utilized cluster analysis to analyze typologies of the social network, however, these analyses are based on different notions. More significantly, the studies that have already been published showed that various support profiles would have varying relationships with outcomes related to MH (Wang et al., 2018). In particular, one study used LPA and discovered that four different profiles of social support have different correlations to elder migrants’ well-being (Burholt et al., 2018).

According to one study, older persons who participated in the social support profile that was locally integrated had a lower risk of dementia than those who participated in the social support profile that was family-dependent (Yang et al., 2020). Researchers discovered different patterns of relations between different profiles of social support and outcomes of MH. The susceptibility of the youth was found to be higher vis-à-vis pessimism and stress irrespective of sources of social support. Social support particularly from family and friends plays a significantly important role in managing MH menaces such as anxiety and depression. LPA directly estimates the membership probability from the model, making it more plausible to examine the outcomes of individual clustering than the conventional method of clustering. LPA being a person-centric strategy helps specifically in identifying different subgroups of people who have similar social support behaviors (Mai et al., 2021). A regression mixed model can be constructed using the LPA model and additional outcome variables to confirm the association between profiles and results. In order to investigate the profiles of sources of social support and the association between these profiles and MH in this study, we did an LPA and used the regression mixture model (BCH technique).

According to research, social support serves as a buffer against depression or the unfavorable consequences of stress (Wilson et al., 2020). It also serves as a buffer between stressful life events and depression. Keeping in view the above facts from the literature the current study assumes that PSS from friends and others positively moderates the relationship between FS and MH it is also hypothesized that PSS from friends and others positively moderates the relationship between FS and coping strategy.

*H5*: Perceived social support from friends and others is to positively moderate the relationship of family support and mental health.

*H6*: Perceived social support from friends and others is to positively moderate the relationship of family support and coping strategy.

Research framework

## 3. Methodology

### 3.1. Sample and data collection procedures

The prospective respondents were the students studying in various universities of China. We selected the Henan province of China for this purpose and 10 large universities of the province were the main target. Because of Covid, it was expected that most of the students stayed at their homes and with their families faced some short of mental disorders. Therefore, to collect data about the study variables, we used a validated survey questionnaire which was circulated to the selected universities’ help desks/information desks for anonymous self-rated responses. As the data collection was related to highly sensitive and personal matters, i.e., MH, family/friends support and coping strategies, and because the population was unknown, we opted for a combination of snow ball and purposive sampling technique (non-probability sampling). In addition to this, for identifying the target respondents, we also sought help from the staff of student affairs.

We targeted 350 deaf students from the concerned universities as the exact no. of students in the target universities remained unknown and not confirmed by the concerned authorities. We therefore, followed the guidelines of various scholars; for example, recommended that “each item should be represented using 5 samples,” who suggested that “sample of 300 will be considered as good,” proposed that “the size of the sample should be 20 times greater than the expected factors” (if factor analysis is to be conducted) and for conducting SEM, *N* = 100–150 is acceptable (Anderson and Gerbing, 1988). Keeping in view the recommendations from these researchers and an average response rate (if any) we determined a sample size of 350 out of which 260 questionnaires were filled and 210 turned out to be valid (response rate 60%). These questionnaires were used for further analysis.

### 3.2. Measurement

All the scales used in this study were adapted from previous researches and were already validated by the scholars. Family support was adapted and its sample item was “I get the emotional help and support I need from my family.” The scales of coping strategies and were adapted from Hamby, and its sample item was “When dealing with a problem, I try to see the positive side of the situation.” The questions of perceived support from friends were adapted, and its sample item was “I can count on my friends when things go wrong.” Likewise the scale items of other’s support were adapted the study of, and its sample item was “There is a special person in my life who cares about my feelings.” Moreover, the measurement questions of MH and its three dimensions, i.e., cognitive, emotional, psychology were adapted and its sample item was “I’ve been feeling optimistic about the future.” All the items were measured at 7 Point Likert scale.

### 3.3. Analysis strategy

To analyze the data, SPSS 21 was used for calculating basic and descriptive statistics while Mplus 7 was applied for hypothesis testing. But prior to moving toward hypothesis testing, we have examined the measurement model in order to ensure the reliability and validity of the constructs, the details of which are discussed in the next section.

## 4. Results

### 4.1. Assessment of measurement model

First of all, researchers analyzed the measurement model keeping in view the requirements of “convergent validity and discriminant validity,” while structural model was assessed in the next step (Anderson and Gerbing, 1988). This research applied Mplus 7, a frontline software which has the potential to handle both normal and non-normal data (Van Der Linden et al., 2017). Mplus 7 output provides the following model fit indices, i.e., “Chi-square,” “root mean square error of approximation (RMSEA),” “comparative fit index (CFI),” “Tucker Lewis index (TLI)” and “standardized root mean square residual (SRMR)” which were used to determine the model fitness. Accordingly, the criterion for model fit indices given by Hu and Bentler (2009) in Table 1 and it can be observed that all the values are lesser than the threshold levels.

**Table 1:** Model fitness.

Model	*χ* ^2^	*χ*^2^/df	CFI	TLI	RMSEA	SRMR
Quality criteria	>0	<5	>0.9	>0.9	≤0.8	≤0.8
**Model**	**761**	**761/512 = 1.4**	**0.956**	**0.952**	**0.048**	**0.067**

*χ*^2^, Chi square value, df, Degree of freedom.

**Figure 1:**
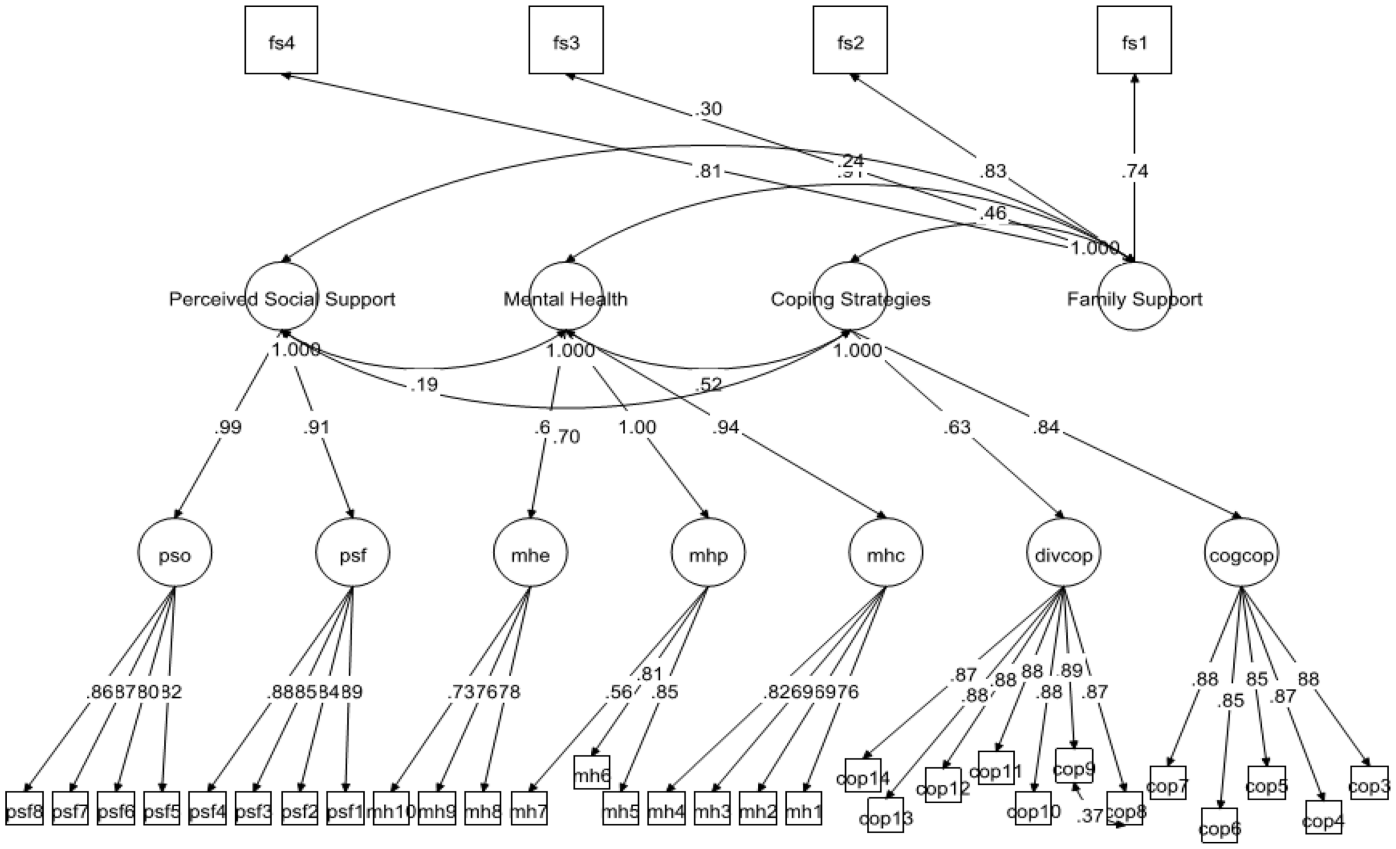
Confirmatory factor analysis.

According to Hair (2009), convergent validity is assessed taking into account the standardized loadings of all the items of the construct/scale. It is important for the scale items to have STDYX >0.5, which is this case. Moreover, the research instrument had 34 items in total and the standardize loadings of all the items ranged from 0.694 to 0.894, which signals the existence of convergent validity (see Figure 1). Additionally, to ensure the internal consistency, convergent validity of the scale, we calculated CR and AVE of the constructs. As the study conducted confirmatory factor analysis, that’s why it was important to compute to these values. However, according to the criteria, for a construct to have internal consistency, it is recommended that the values of CR to remain at least 0.7 or greater, while the values of AVE should at least pass the minimum threshold level of 0.5 (Fornell and Larcker, 1981). The results in Table 2 confirmed that the scale met the criteria of internal consistency.

**Table 2:** Factor loadings, composite reliability and convergent validity.

Latent variable	*π*	CR	AVE
Family support by		**0.89**	**0.68**
FS1	0.773		
FS2	0.834		
FS3	0.910		
FS4	0.809		
Cognitive coping strategy by		**0.93**	**0.75**
COGCOP1	Deleted		
COGCOP2	Deleted		
COGCOP3	0.878		
COGCOP4	0.873		
COGCOP5	0.850		
COGCOP6	0.846		
COGCOP7	0.878		
Diversion coping strategy		**0.95**	**0.77**
DIVCOP1	0.866		
DIVCOP2	0.894		
DIVCOP3	0.879		
DIVCOP4	0.878		
DIVCOP5	0.876		
DIVCOP6	0.881		
DIVCOP7	0.873		
Mental health by (Cognitive)		**0.83**	**0.55**
MHC1	0.758		
MHC2	0.694		
MHC3	0.695		
MHC4	0.825		
Mental health (Psychological)		**0.79**	**0.56**
MHP5	0.849		
MHP6	0.806		
MHP7	0.555		
Mental health (Emotional)		**0.80**	**0.57**
MHE8	0.775		
MHE9	0.761		
MHE10	0.726		
Friends support		**0.92**	**0.75**
PFS1	0.889		
PSF2	0.838		
PSF3	0.850		
PSF4	0.878		
Others support		**0.90**	**0.70**
PSO1	0.825		
PSO2	0.797		
PSO3	0.871		
PSO4	0.860		

*N* = 210, AVE, average variance extracted; π, standardized loadings; FS, Family support; COGCOP, cognitive coping; DIVCOP, diversion coping; MHC, cognitive aspect of mental health; MHE, emotional aspects of mental health; MHP, psychological aspects of mental health; PSF, perceived social support from friends; PSO, perceived social support from others. The bold values indicate the results for corresponding statistics for the whole variable.

### 4.2. Determining correlation and discriminant validity

In order to determine whether the scale has discriminant validity or not, comparing the squared root of the AVE with the correlation coefficient was essential (Fornell and Larcker, 1981). For this purpose, diagonal bolded values were compared with the off-diagonal values in Table 3. It can be observed that the diagonal values remained greater than the correlation coefficients, which depicted a strong evidence of validity.

**Table 3:** Correlation and discriminant validity.

Variables	FS	COP	MH	PSS
FS	**0.825**			
COP	0.464	**0.744**		
MH	0.241	0.521	**0.88**	
PSS	0.303	0.703	0.1 92	**0.97**

All values are significant **p < 0.05; FS, Family support; COP, Coping strategies; MH, Mental Health; PSS, Perceived social support from others and friends.

In addition to this, we calculated variance inflation factor to test the existence of multicollinearity. The analysis of the multicollinearity showed that the VIF values were <3 which confirmed the model did not have the issue of multicollinearity.

### 4.3. Measuring descriptive statistics

The variability of the data was assessed by analyzing the mean values and standard deviation from the mean. The analysis presented in Table 4 revealed the MEAN values of the variables and their standard deviation.

**Table 4:** Mean and standard deviation.

Variables	Mean	Std.
Family support (FS)	5.01	1.023
Coping strategies (COP)	5.17	0.587
Perceives social support from friends and others (PSS)	6.38	0.819
Mental health (MH)	5.63	0.930

Std., standard deviation of the construct; FS, family support; COP, coping strategies.

The mean scores of the variables ranged from 5.01 of FS to 6.38 of PSS whereas the standard deviation ranged from 0.587 of COP to 1.023 of FS. The values of standard deviation remained well within prescribed range and the data was found to be good to enough to be handled using Mplus (Van Der Linden et al., 2017). To compliment this, we also calculated Skewness/Kurtosis to make assessment about the normality of the data. It was found that the values of both the statistical measures were well within acceptable range. Table 4 placed above provides variable specific details about normality and data variability.

### 4.4. Test of spurious correlation/(CMV)

As the researcher collected survey data at a single point of time and from the same students who suffered some sort of MH issues during COVID-19, therefore, it was important to make sure that the correlation between the said variables was not spurious. For this purpose, Harman’s single-factor was applied to assess the values of CMV. As shown in the Table 5, the single factor explained 33.269% of variance, which was far below the standard limit of 49%. If the result demonstrate that less than half (50%) of the variance is explained by the factor, then it is believed that CMB is not a cause of concern.

**Table 5:** Common method variance analysis.

Factor	Initial eigen values			Extraction sums of squared loadings		
	Total	Variance	Cumulative	Total	Variance	Cumulative
1	12.641	33.269%	33.269%	12.642	33.269%	33.269%

### 4.5. Hypothesis testing

The Following section presents the results of direct association, indirect association and the role of the moderator, respectively.

**Figure 2:**
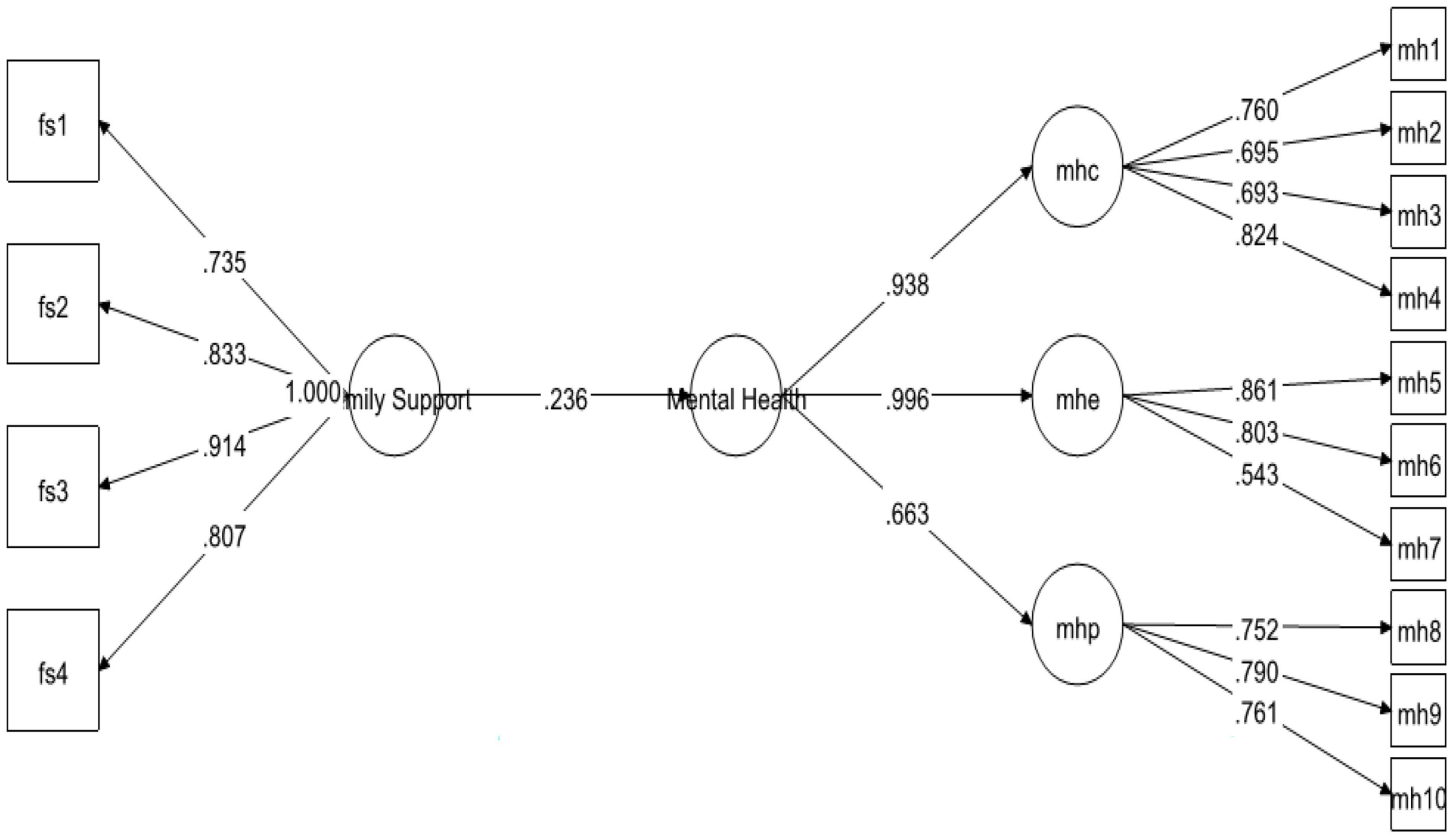
Direct relationship between FS and MH.

**Figure 3:**
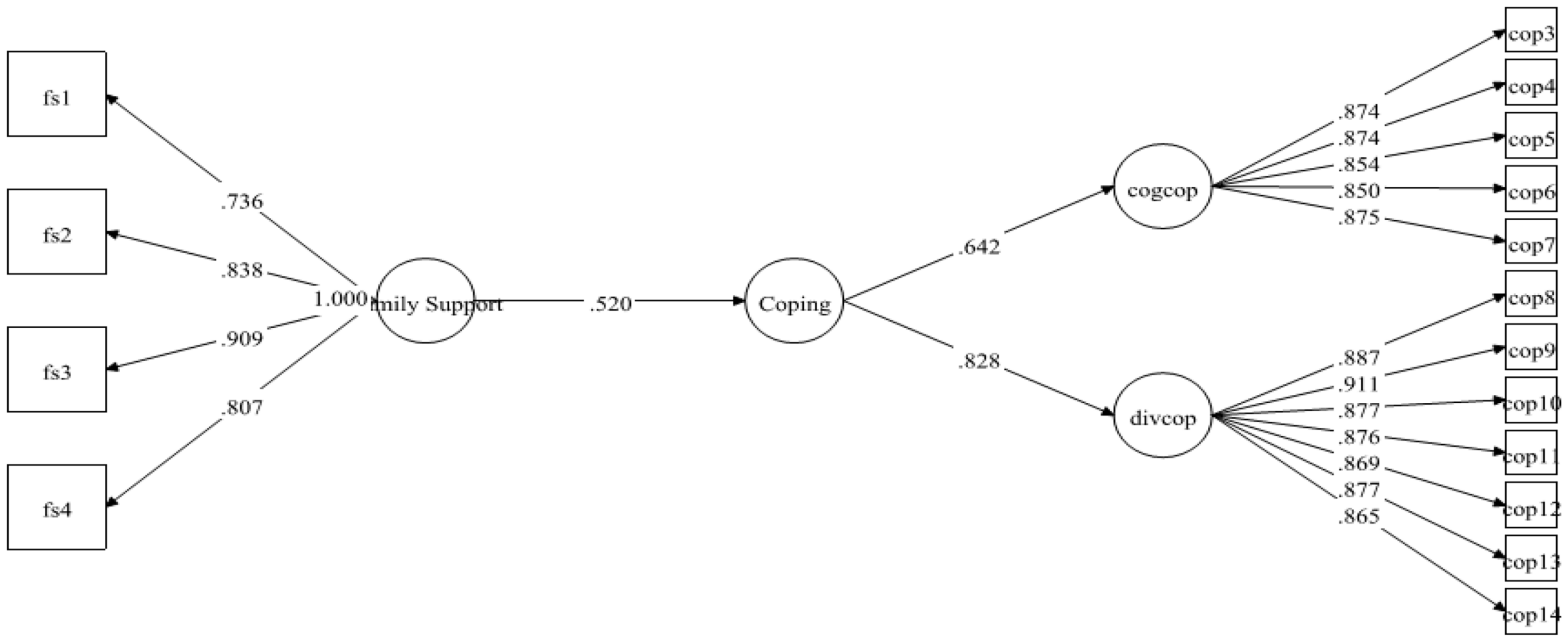
Direct relationship between FS and coping strategy.

**Figure 4:**
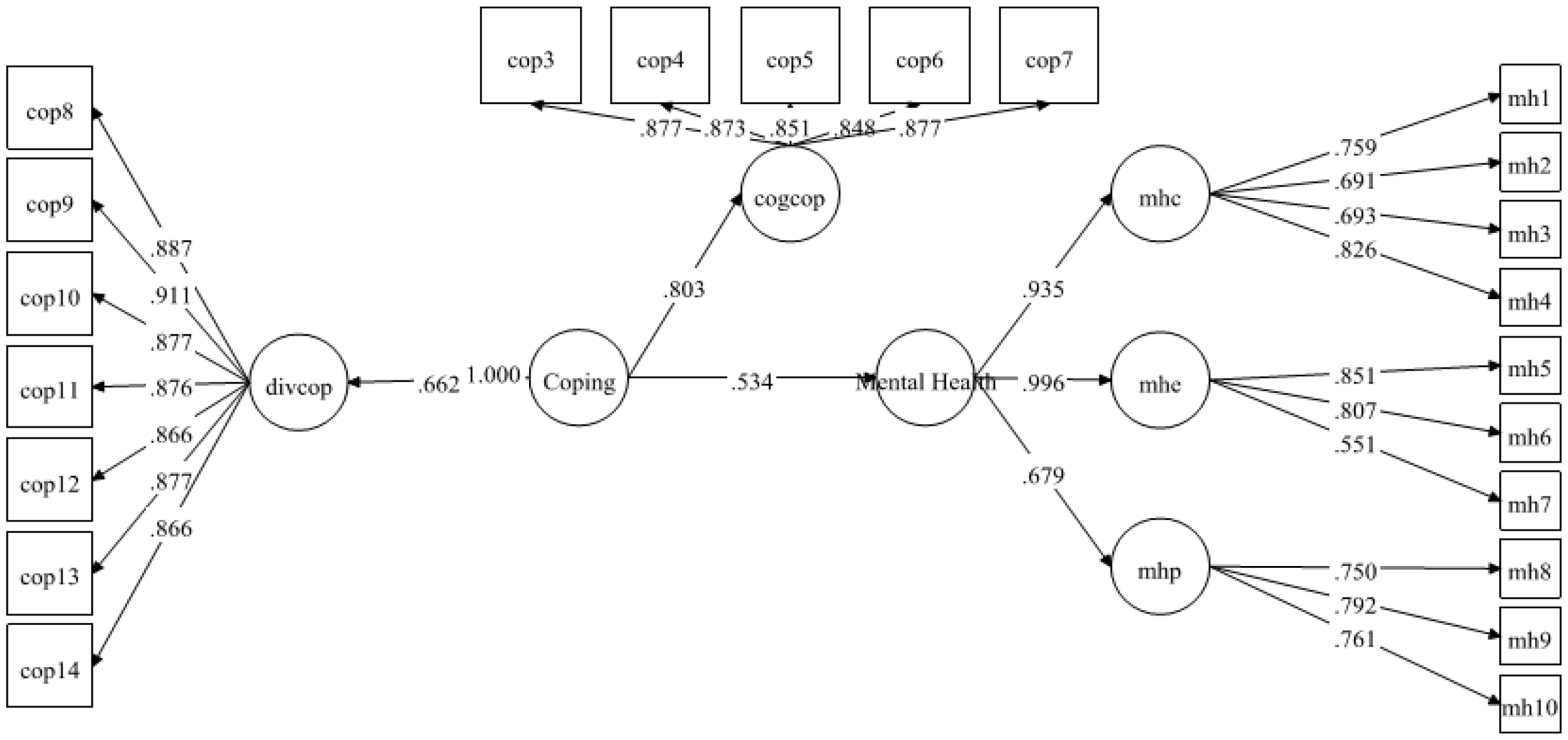
Direct relationship between coping strategy and MH.

Table 6 contains information about the hypotheses of direct relationship. As pointed out by Henseler et al. (2009), the standardized coefficients are the same as the regression coefficients, that’s why, the decision about the hypothesis were made based upon the standardized coefficients and their significance (value of ps) provided in Mplus 7 output. The results demonstrated that FS was strongly positively linked to MH with *β* = 0.236 and was significant at 0.00. Therefore, H1 was supported (see Figure 2). Likewise, H2 examined the relationship between FS and coping strategies. The results exposed that FS positively predicted coping strategies with *β* = 0.520 and *p*-value 0.00. Hence H2 was also found to be supported (see Figure 3). Finally, for direct relationship, the link between coping strategy and MH was also determined. The researcher found out that “coping strategy” strongly predicted MH with *β* = 0.534 and *p*-value 0.000. Therefore, H3 was supported as per the prediction of the researcher (see Figure 4).

**Table 6:** Hypothesis testing for direct relationship.

Hypothesis	Β-value direct	Value of *p*	Outcomes
H1: Family Support—Mental Health	0.236	0.001	Supported
H2: Family Support—Coping Strategy	0.520	0.000	Supported
H3: Coping Strategy—Mental Health	0.534	0.000	Supported

**Figure 5:**
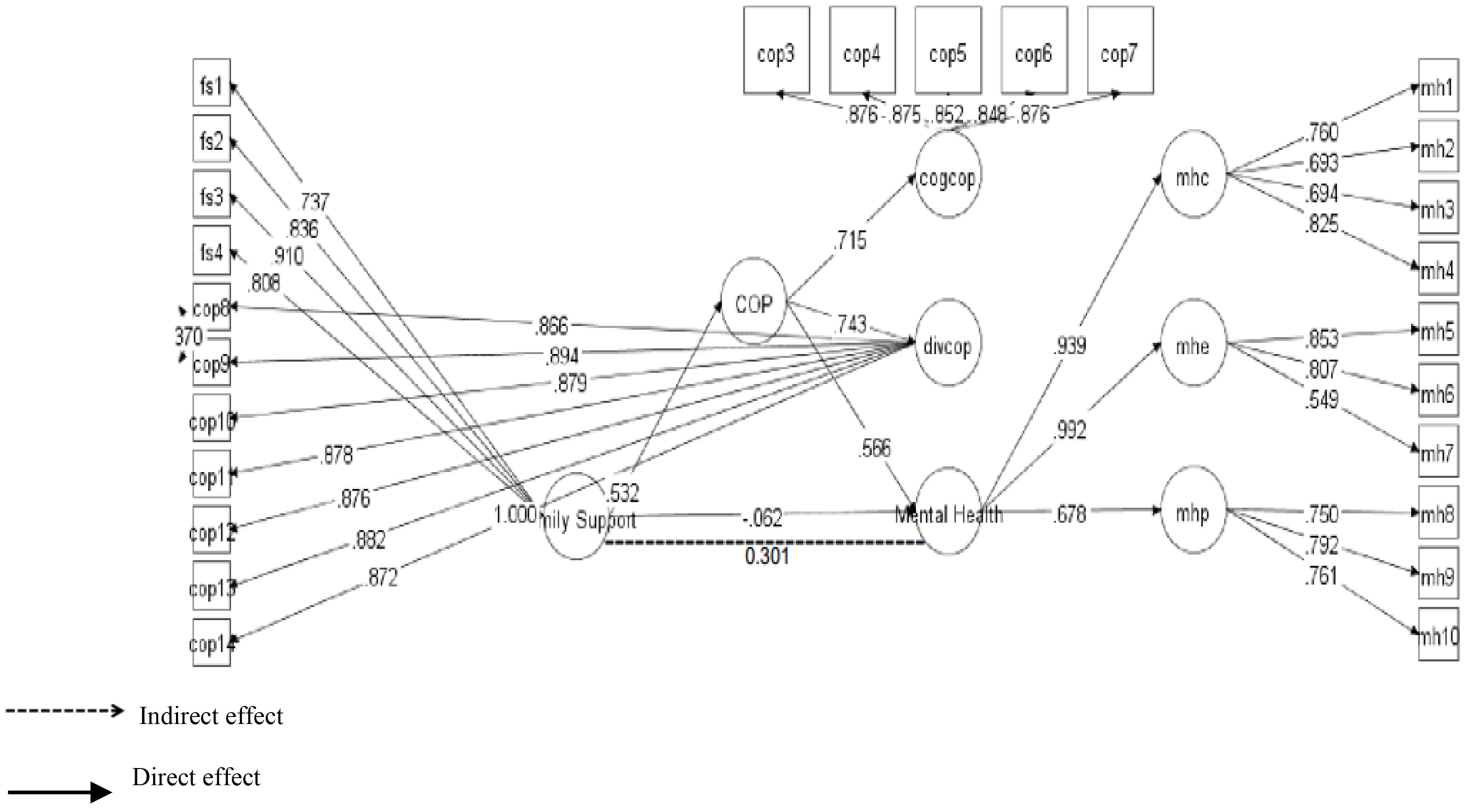
Mediation of coping strategy between FS and MH.

Results in Table 7 were about H4 which was related to the mediation analysis. As per the requirement, mediation analysis was performed to confirm whether the intervening/mediating variable (MV) enhanced the impact of IV to the DV (Hair, 2011). The researcher opted for, “bootstrapping procedures” to test the significance of the mediation path (Preacher and Hayes, 2004; Preacher et al., 2010). The analysis revealed that specific indirect effect was significant at 0.05. The STDYX of SIE (specific indirect effect) was *β* = 0.301 and (*p* = 0.00) with a 95% CI [0.154–0.148]. Because the upper CI and lower CI values did not include zero, therefore, it was confirmed that “coping strategies” positively and significantly mediated the relationship of “family support” and “MH.” Thus H4 was supported (see Figure 5).

**Table 7:** Hypothesis testing for mediation.

Hypothesis	*β*-value SIE	95% SIE	Outcome
H4: Mediation of COP between FS—MH Relationship	0.301 (0.00)	0.154–0.448	Supported

All values are significant **p < 0.05. SIE, specific indirect effect; Stdyx, standardized beta coefficient; CI, confidence interval; COP, coping strategies; MH, mental health; PSS, perceived social support from others and friends.

**Figure 6:**
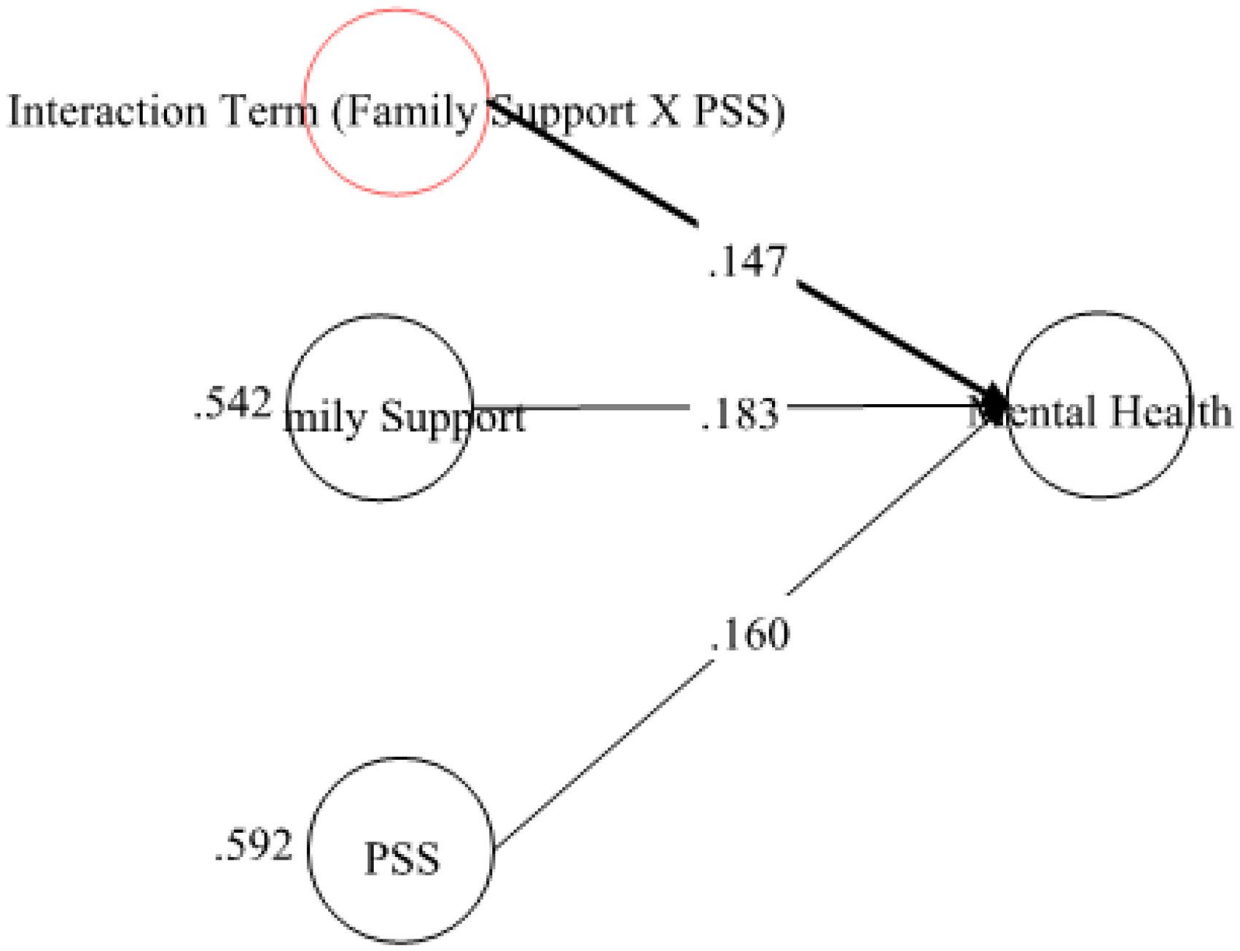
Theoretical model for mediation of coping strategy between FS and MH.

**Figure 7:**
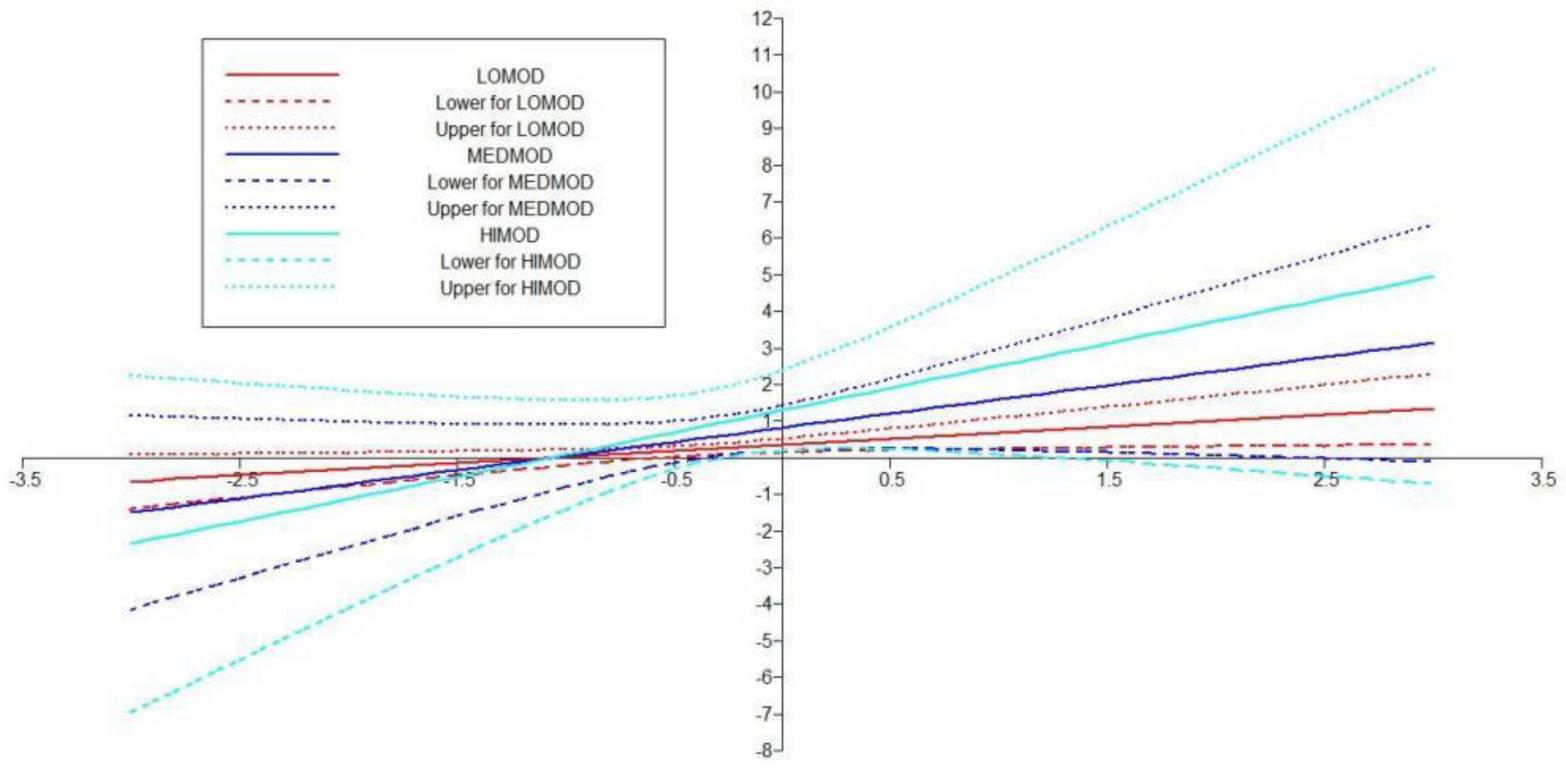
Interaction plott showing the moderating effect of PSS on FS—MH relationship.

Fore investigating the moderating role of PSS, the study first examined its role on the relationship of FS and MH. Therefore, in order to proceed with the analysis, researcher applied “Xwith” technique for creating the interaction term between concerned IV-MV (Van Der Linden et al., 2017) and for assessing the effect of interaction term (see Figures 6, 7). These results exposed that H5 was not supported as the Table 8 indicated that the interaction effect of PSSXFS (moderating variable * IV) was *β* = 0.147 (*p* = 0.228) with a 95% CI [−0.054 to 349].

**Table 8:** Hypothesis testing for moderation analysis.

Hypothesis	Direct relationship	Moderation (Interaction effect)	95% CI	Outcomes
H5: PSS positively moderates the relationship of family support and mental health	FS—MH = 0.183 (0.020)	0.147 (0.228)	[−0.054 to 349]	Not supported
	PSS—MH = 0.160 (0.054)			
H6: PSS positively moderates the relationship of family support and coping strategies	FS—COP = 0.263 (0.00)	0.357 (0.002)	[0.135 to 0.391] [0.536 to 0.855]	Supported
	PSS—COP = 0.695 (0.00)		[0.171 to 0.544]	

FS, family support; COP, coping strategies; MH, mental health; PSS, perceived social support from others and friends; Stdyx, standardized beta coefficient; CI, confidence Interval.

**Figure 8:**
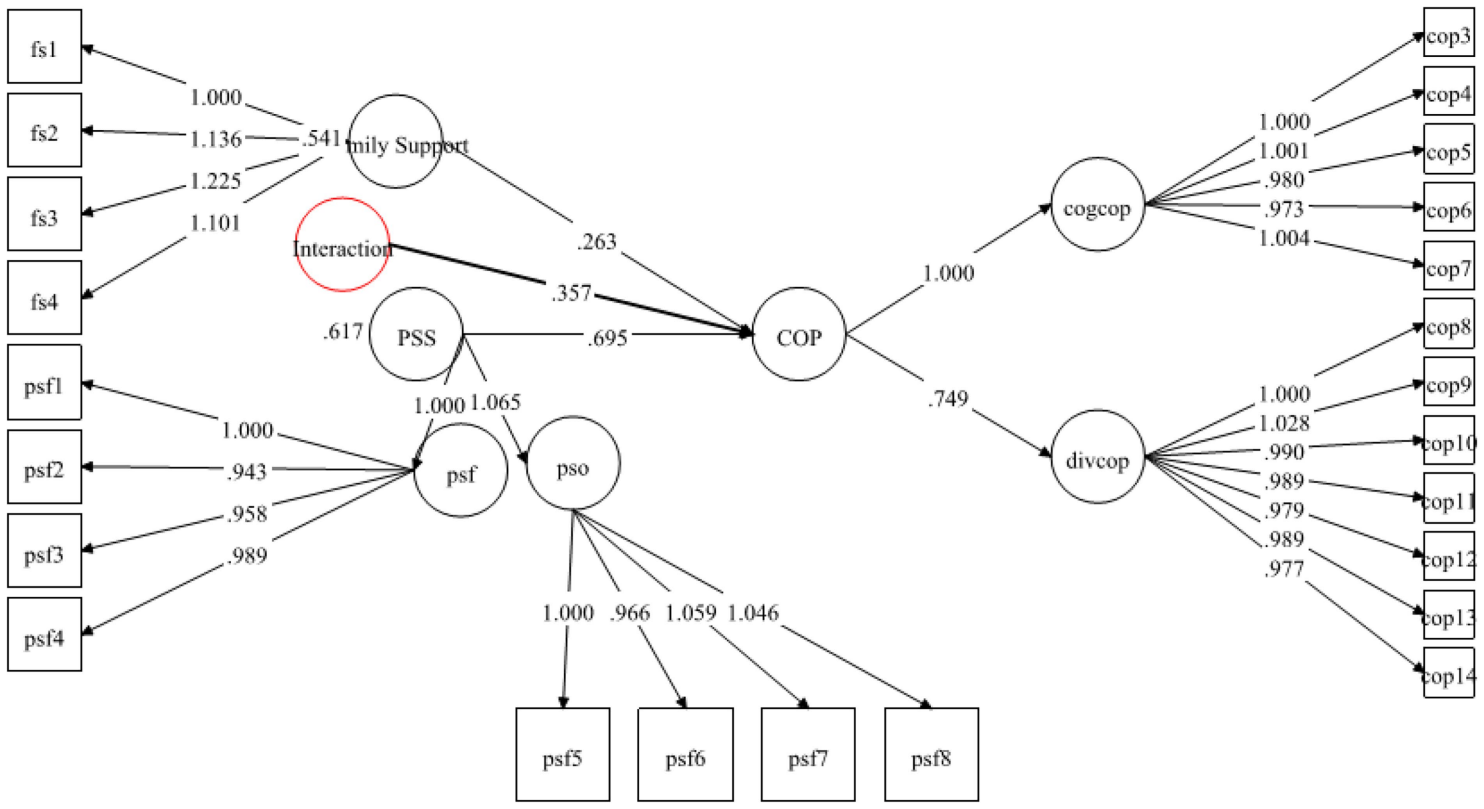
Moderating effect of PSS on FS—COP relationship.

**Figure 9:**
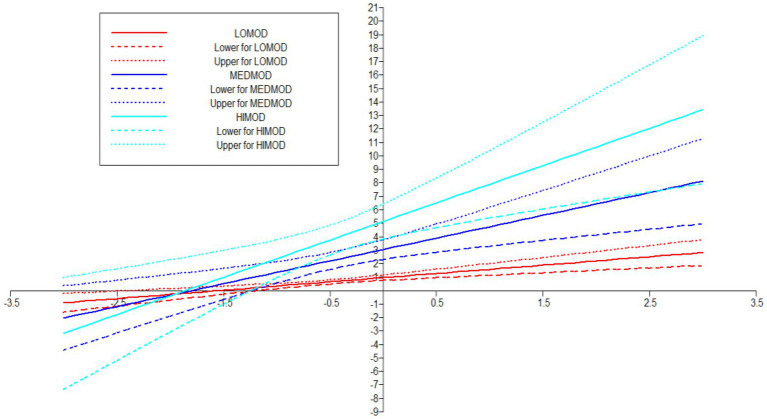
Interaction platt showing the moderating effect of PSS on FS—COP relationship.

In Hypotheses 6, it was predicted that PSS will positively moderate the relationship of FS and COP. The results in Table 8 indicated that the direct effect of FS on COP was *β* = 0.263 (*p* = 0.00) and its 95% CI [0.135–0.391], the direct effect of moderating variable PSS on COP was *β* = 0.695 (*p* = 0.00) with 95% CI [0.536–0.855] and the interaction effect of FSXPSS (moderating variable * IV) was *β* = 0.357 (0.002) with a 95% CI [0.171–0.544] (see Figure 8). Additionally, Simp-Slope analysis revealed that this moderation effect was greater when the values of PSS were higher and positive, whereas, the effect was lower and positive when the values of PSS were lower. These values implied that H6 was supported (see Figure 9).

## 5. Discussion

Around the world, 10%–20% of teenagers experience MH issues. The vulnerability of teenagers during the COVID-19 epidemic is likely to have an impact on this figure. Poor MH leads to negative outcomes such as suicidal tendencies, behavioral issues, and emotional abnormalities, therefore, the need for research in this regard to eliminate or lessen the impact of poor MH is paramount. As the MH of young people has already been in tattered shape globally, COVID-19 has made the case even more precarious. The need to conduct quality research to combat MH issues in the wake of a pandemic of COVID-19’s size has increased manifolds. Keeping in view these facts, we identified our research topic, research questions and including coping strategies and their relationships with post COVID-19 MH of the students.

The current article discussed the literature on family and PSS and MH of the individuals after COVID-19 pandemic keeping in view the stress-health theory. The literature review gave us an insight into the research that has already studied coping strategies for MH. The studies have shown that the pandemic and the factors related to it like quarantine, social distancing, and travel restrictions have been proven stressful for the students and other communities. The stress and anxiety produced by the situations like school closures, unemployment, insufficient health facilities, and uncertainties related to study, work, and personal life have negatively affected the MH and physical well-being of the people (Pfefferbaum and North, 2020). Keeping in view the importance of coping strategies after the pandemic we selected a framework that could serve the literature both theoretically and practically.

Previous studies on coping strategies and PSS have not studied their roles as mediators and moderators in a single framework. The current study fills this literature gap and first of all, it assumed that FS is positively associated with MH. The data analysis showed that FS is significant and strongly positively linked to MH with *β* = 0.236, thus supporting the H1 (see Figure 2). Likewise, H2 examined the relationship between FS and coping strategies. The results revealed significant values for both of these variables thus H2 has been accepted as well (see Figure 3). Finally, for a direct relationship, the link between coping strategy and MH was determined. The researcher found out that coping strategy strongly predicted MH by showing significant values, the prediction of the researcher was supported by the results of the analysis, and H3 was accepted (see Figure 4). The mediation analysis was done to check the acceptance or rejection of H4. It has been explained in the results section earlier that mediation analysis was performed to confirm whether the intervening/mediating variable (MV) enhanced the impact of IV to the DV. The analysis was done by using “bootstrapping procedures” which showed that the upper CI and lower CI values do not include zero, therefore, it is confirmed that “coping strategies” positively and significantly mediated the relationship of “FS” and “MH.” The results supported the acceptance of the H4 (see Figure 5). The analysis to investigate H5 was carried out further, and the moderating role of PSS “Xwith” technique was used. The results obtained from the analysis have shown that H5 was not accepted, the analytical values for H5 have been explained in the results section. One of the plausible reasons for this insignificant moderation effect could be the either lesser social support received by the students in the times COVID-19 from friends and others. Secondly, due to social restrictions, no one from the social circle of the students would have been willing to extend their support for their friends who faced MH issue. Thirdly, students struggled with MH placed more value on FS and remained open to it instead of relying on the support of friends and others. Similarly, the results in Table 7 showed the values for the direct effect of FS on COP and the values for the direct effect of moderating variable PSS on COP (H6). Additionally, Simp-Slope analysis was carried out to reveal the moderation effect of PSS, the results of which indicate that H6 was accepted (see Figure 9).

Our research findings show that the hypotheses H1, H2, H3, H4, and H6 were accepted which means that FS positively influences MH and is positively associated with coping strategy. Then the findings show that coping strategy positively influences MH and it mediates the relationship between FS and MH. Further, our finding has shown that PSS positively moderates the relationship of FS and coping strategy which is consistent with the research findings which revealed that PSS mitigates the negative effects of stress and fosters psychological (Huang and Zhang, 2021). However, our findings do not accept that PSS positively moderate the relationship of FS and MH.

## 6. Conclusion

The COVID-19 pandemic and the multifaceted response strategies to curb its spread both have devastating effects on mental and emotional health. Social distancing, and self-isolation have impacted the lives of students. These impacts need to be identified, studied, and handled to ensure the well-being of the individuals, particularly the students. Therefore, current study aimed to analyze the role of coping strategies, family support, and social support in improving the MH of the students by collecting evidence from post COVID-19. Data was collected from the students studying in Chinese universities. A survey questionnaire was designed to collect data form 210 students. Descriptive statistics were calculated using SPSS 21 while hypothesis testing was carried out using Mplus 7. The results highlighted that the coping strategy a person uses to deal with challenging or complicated unfavorable situations is crucial since it will have an impact on their psychosocial outcomes, especially their MH. Positive and negative coping are opposites of one another. People who used mostly constructive coping mechanisms had less emotional disturbance than those who used destructive coping mechanisms (Budimir et al., 2021). Additionally, the study highlighted the significant role of family members and how they can support the fight against mental disorders. Results have also exposed the fact that students not only need FS but also the support from friends and others in order to develop effective coping strategies to deal with the MH issues stress caused by various factors. Therefore, in the future, we propose to investigate the rationale for expanding the model of cognitive vulnerability by including PSS. Resulting in fresh questions about the potential and necessity of moving forward not only toward an integrated model but also etiological models as well as novel opportunities for research and practical applications.

## 7. Implications

Our research has significant ramifications for medical practice, research institutions, and health policy. Academic institutions should first become more aware of any additional needs and any MH issues that their students may have. Future studies should involve participants from other nations and ethnic groups because COVID-19 control strategies and the scope of outbreaks varied among nations. The coping techniques we incorporated in the survey were chosen in a way that students might find interesting to engage with, but the list was not comprehensive, and well-liked techniques may not always be the best ways to guard against negative effects on MH. A wider range of coping mechanisms should be the subject of more research to determine their effectiveness. In conclusion, the effects of COVID-19 on students’ MH have been underappreciated. We enjoin teachers, higher education institutions, and specialists in MH to give their pupils the proper support during the pandemic.

Giving students instruction to help them develop self-efficacy and providing them with useful skills to cope with challenges may assist them in better managing the heightened stress that entails COVID-19. As it has been noticed that strong and effective coping strategies were highly useful in helping students manage their stress. The role of MH practitioners needs to be recognized by the administrators so that they can help those students that require MH assistance. Students’ ability to cope with stress and build social support may help them avoid the negative effects of the corona virus epidemic on their mental and psychological health. As a result, by implementing theory-tested interventions or programs, families, friends, and educators should promote psychological resilience and strengthen positive coping mechanisms among students. These interventions could be offered in creative methods, such as webinars, online seminars, and on-demand movies, due to limits like social isolation and lockdown measures.

Students’ endurance and confidence are improved through inter professional, debriefing programs and online cognitive behavioral therapy (Schmutz, 2022). Additionally, enhancing social support may provide people a feeling of greater mental stability, lowering their worries and anxieties and allowing them to operate normally during the epidemic. Students’ morale will rise and their MH will be maintained if they are encouraged to openly discuss their experiences and difficulties in their education following COVID-19.

## Data availability statement

The original contributions presented in the study are included in the article/supplementary material, further inquiries can be directed to the corresponding authors.

## Ethics statement

The studies involving human participants were reviewed and approved by, the Department of Educational Psychology and Counselling, Faculty of Education, University of Malaya, Kuala Lumpur, Malaysia. The patients/participants provided their written informed consent to participate in this study.

## Author contributions

CY and HG worked on conceptualization. NW and QW worked on data collection. YL and EW worked on writing the draft. The authors read and agreed to the submitted version of the manuscript.

## Funding

This study was funded by Research on the Construction of the Training System for Outstanding Special Teachers in the New Era-The key project of Teacher Education Curriculum Reform of the Education Department of Henan Province in 2019 (no. 2019-JSJYZD-058).

## Conflict of interest

The authors declare that the research was conducted in the absence of any commercial or financial relationships that could be construed as a potential conflict of interest.

## Publisher’s note

All claims expressed in this article are solely those of the authors and do not necessarily represent those of their affiliated organizations, or those of the publisher, the editors and the reviewers. Any product that may be evaluated in this article, or claim that may be made by its manufacturer, is not guaranteed or endorsed by the publisher.
